# Novel technique using metal clip and dental floss facilitates difficult biliary cannulation in Billroth II gastrectomy

**DOI:** 10.1055/a-2173-7348

**Published:** 2023-10-06

**Authors:** Zhichu Qin, Jianlong He, Zhihe Deng, Yan Qin, Junlian He, Fenhua Ye, Lihao Wu

**Affiliations:** 1 Department of Gastroenterology, The First Affiliated Hospital of Guangdong Pharmaceutical University, Guangzhou, China; 2 Research Center for Engineering Techniques of Microbiota-Targeted Therapies of Guangdong Province, Guangzhou, China

We report the case of a 79-year-old woman with a previous Billroth II gastrectomy for gastric cancer who presented with jaundice (total bilirubin 120.72 mg/dL, direct bilirubin 96.3 mg/dL) and abdominal pain. Magnetic resonance pancreaticobiliary imaging demonstrated extrahepatic bile duct dilation and a distal common bile duct (CBD) stone (1.2 × 1 cm). Endoscopic retrograde cholangiopancreatography in patients with Billroth II anatomy is associated with low success rates of selective access to the afferent loop and cannulation of the CBD owing to the inverted position, as well as a high incidence of complications 1 2 . For the current case, a forward-viewing colonoscope with a clear cap was chosen over a duodenoscope to decrease the difficulty of entry and limit potential adverse events, such as perforation 3 .

On initial inspection, the major papilla was located on the left side of the screen and was inverted compared with normal anatomy, making cannulation difficult ( Fig. 1, Video 1 ). A metal clip was employed to hold the ampullary mucosa above the major papilla, and dental floss traction on the metal clip fixed the papilla in position, improving the CBD axis and maximizing traction for biliary cannulation ( Fig. 2 , Video 1 ). Cannulation was then achieved without incident.

**Fig. 1:**
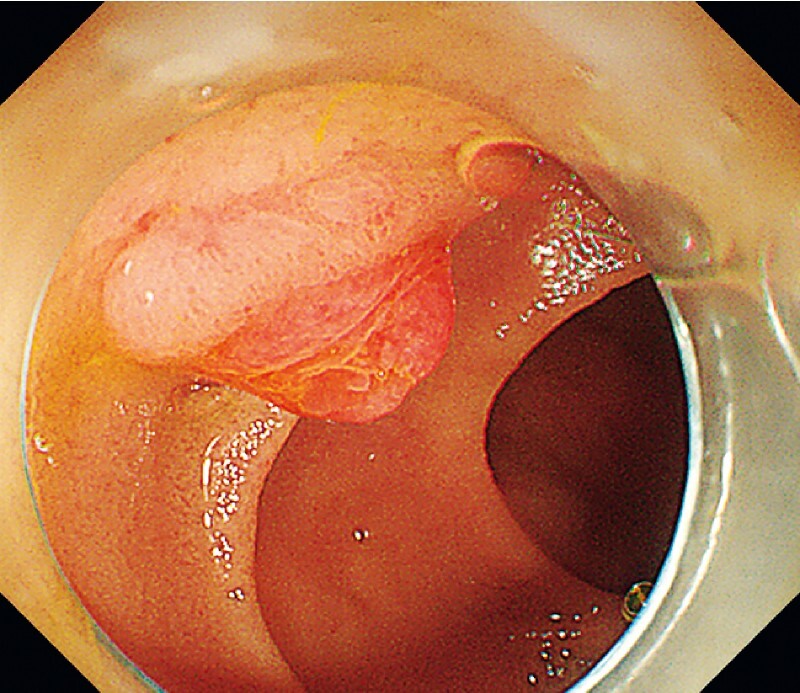
The major papilla was inverted compared with the normal anatomical position.

**Video 1** Metal clip and dental floss as an alternative technique for treating difficult biliary cannulation in Billroth II anatomy.


**Fig. 2:**
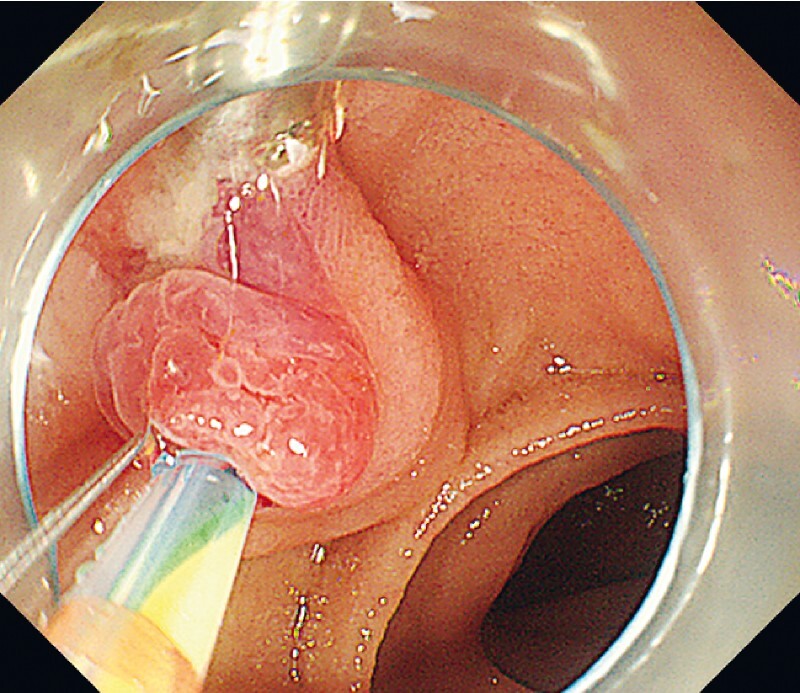
The metal clip held the ampullary mucosa above the major papilla, and dental floss traction on the metal clip fixed the papilla in position, facilitating biliary cannulation.

Fluoroscopy evaluation revealed a dilated CBD with a large biliary stone ( Fig. 3 ). Endoscopic large-balloon dilation up to 10 mm was performed 4 . The stone was removed without any problems using a stone basket. Final radiography indicated no residual stones ( Fig. 4 , Video 1 ). A plastic stent was placed into the CBD. The metal clip was removed after the operation. No adverse events occurred.

**Fig. 3:**
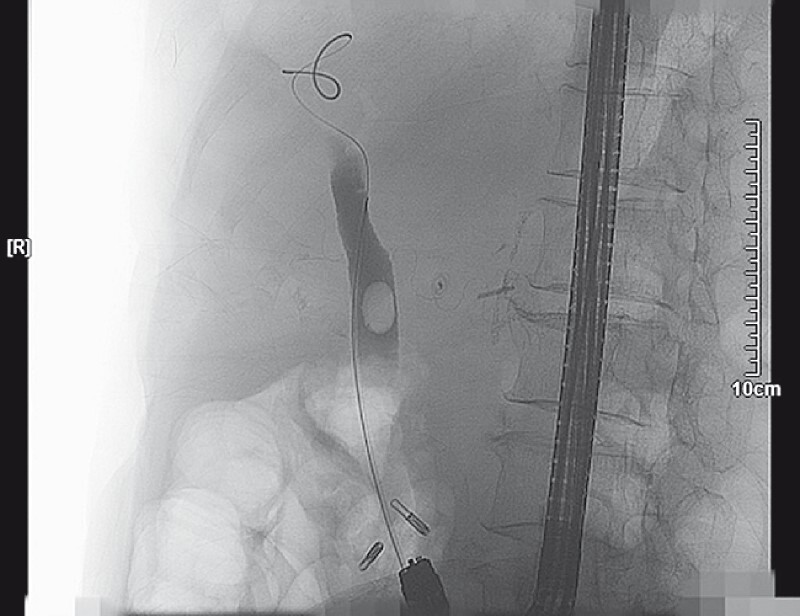
A large stone was discovered in the common bile duct.

**Fig. 4:**
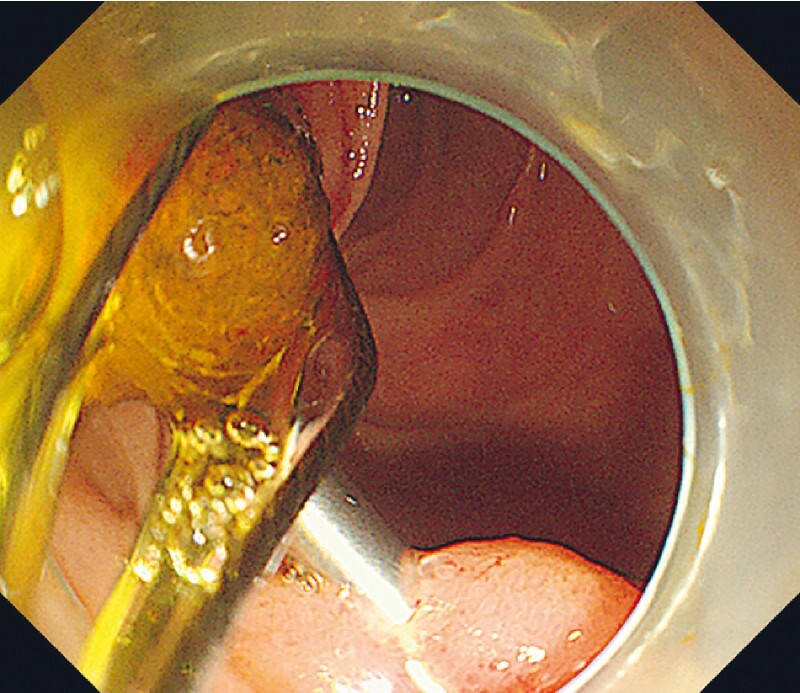
The stone was removed with a stone basket.

The pulling force of the metal clip and dental floss can change the orientation of the major papilla, allowing swift and successful cannulation, which is especially useful in patients with surgically altered gastrointestinal anatomy.

Endoscopy_UCTN_Code_CCL_1AZ_2AI

## Notes

Endoscopy E-Videos

https://eref.thieme.de/e-videos
*E-Videos* is an open access online section of the journal *Endoscopy* , reporting on interesting cases and new techniques in gastroenterological endoscopy. All papers include a high-quality video and are published with a Creative Commons CC-BY license. Endoscopy E-Videos qualify for HINARI discounts and waivers and eligibility is automatically checked during the submission process. We grant 100% waivers to articles whose corresponding authors are based in Group A countries and 50% waivers to those who are based in Group B countries as classified by Research4Life (see: https://www.research4life.org/access/eligibility/ ). This section has its own submission website at https://mc.manuscriptcentral.com/e-videos
